# Characterisation and Pathogenicity of *Aspergillus tamarii* Causing Banana Fruit Rot

**DOI:** 10.21315/tlsr2021.32.3.10

**Published:** 2021-09-30

**Authors:** Latiffah Zakaria, Chai Yan Yan, Masratul Hawa Mohd, Nur Amalina Kamaruddin, Nurul Farizah Azuddin

**Affiliations:** School of Biological Sciences, Universiti Sains Malaysia, 11800 USM Pulau Pinang, Malaysia

**Keywords:** Banana Fruit Rot, *Aspergillus tamarii*, Postharvest, Pathogenicity, Phylogenetic, Reput Buah Pisang, *Aspergillus tamarii*, Lepas Tuai, Kepatogenan, Filogenetik

## Abstract

Banana fruit rot is a common postharvest disease of the banana fruit. The appearance of rot symptoms on the surface of the fruits reduces the quality and marketability of banana. From rot lesions on banana fruits, three *Aspergillus* isolates were isolated. Based on morphological characteristics and sequences of Internal Transcribed Spacer, β-tubulin and calmodulin, the isolates were identified as *A. tamarii*. Pathogenicity tests of the isolates, conducted using mycelial plugs with wounded and unwounded treatments, showed *A. tamarii* as the pathogen of banana fruit rot. Rot symptoms were highly severe on wounded banana fruits compared to unwounded fruits, and therefore, wounded banana fruits are more susceptible to *A. tamarii* infection. To the best of our knowledge, this is the first report of *A. tamarii* as a causal pathogen of banana fruit rot. This study indicated *A. tamarii* is one of postharvest rot pathogens of banana.

## Abstract (fr)

Reput buah pisang merupakan penyakit lepas tuai yang lazim pada buah pisang. Kemunculan gejala reput pada permukaan buah mengurangkan kualiti dan kebolehpasaran buah pisang. Dari lesi reput pada buah pisang, tiga pencilan *Aspergillus* telah dipencilkan. Berdasarkan ciri-ciri morfologi dan jujukan Trankripsi Penjarak Dalaman (ITS), β-tubulin dan kalmodulin, pencilan tersebut dikenal pasti sebagai *A. tamarii*. Ujian kepatogenan yang dilakukan menggunakan palam miselium dengan rawatan luka dan tidak luka, menunjukkan *A. tamarii* merupakan patogen reput buah pisang. Gejala reput amat teruk pada buah pisang yang luka berbanding buah yang tidak luka. Oleh itu, buah pisang yang luka lebih mudah dijangkiti *A. tamarii*. Pada pengetahuan kami, ini adalah laporan pertama *A. tamarii* sebagai patogen penyebab reput buah pisang. Kajian ini menunjukkan *A. tamarii* merupakan salah satu patogen reput pisang lepas tuai.

**Highlights**

- *Aspergillus tamarii* (*A. tamarii*) from banana fruit rot was identified molecularly based on Internal Transcribed Spacer, β-tubulin and calmodulin sequences.
- *A. tamarii* is a pathogen of banana fruit rot, causing highly severe infection on wounded banana fruit.
- Combined effect of fruit rot pathogens including *A. tamarii* has the potential to severely infect bananas, which affects their quality and marketability.

Banana (*Musa* spp.) is one of the most important fruit crops in Malaysia with planted area of 30,455 ha and production at 330,957 tonne metrics in 2018 (Ministry of Agriculture and Agro Based Industry, Malaysia 2018). The fruit is also the world’s most popular and widely consumed fruits due to their nutritional value and it is available all year round (Khazanah Research Institute 2019).

Like any other fruit crops, banana is susceptible to postharvest diseases, commonly due to the effects of physiological states and poor handling of the harvested banana. Banana is a climacteric fruit that ripen after harvest and at this stage respiration is rapid and massive production of ethylene. At this stage, the fruits are also susceptible to infection by postharvest pathogens (Turner 2001). Other factors contributing to the occurrence of banana postharvest disease are poor disease management practices in the farms which can lead to physical injuries and unclean packing houses (Nelson 2008), long transportation and storage (Lassois *et al*. 2010). Postharvest disease of banana can also occurs at the selling point, such as at the grocery store or after purchase (Coates & Johnson 1997). Due to postharvest diseases, 10%–30% losses have been reported from total yield (Agrios 2005).

The appearance of rot symptoms on the banana fruits causes the bananas to have a lower market value, although the infected fruits are still safe for human consumption. However, the shelf life and fruit quality are reduced (Nelson 2008).

*Aspergillus* species are not regarded as major plant pathogen; nevertheless, several species can cause rotting on various types of crops. The most common species associated with plant diseases as well as contaminated agricultural products are *Aspergillus niger* (*A. niger*) and *A. flavus* followed by *A. parasiticus*, *A. ochraceus*, *A. carbonarius* and *A. alliaceus*. *Aspergillus* species infected crops during pre-harvest, after harvest, during processing, handling, storage and marketing (Perrone *et al*. 2007).

In the present study, three isolates of *Aspergillus* (BPMF17, MF9 and MF24) were recovered from banana fruits showing symptoms of fruit rot. The banana fruits were purchased from a fruit stall in Pulau Pinang, Malaysia. Thus, the objectives of the present study were to identify the *Aspergillus* isolates using morphological and molecular characteristics, and to conduct pathogenicity test to determine whether the isolates are the causal pathogen of banana fruit rot.

*Aspergillus* isolates MF9 and MF24 were isolated from the banana *Mas* cultivar, and BPMF17, from *Berangan* cultivar. The isolates were identified based on colony appearance on malt extract agar (MEA), Czapek yeast autolysate agar at 25°C (CYA25), and Czapek yeast autolysate agar at 37°C (CYA37) as well as the characteristics of conidiophore and conidia (Samson *et al*. 2010).

The colony characteristics of the three isolates were similar. The upper colonies on MEA [Fig. 1(A1)], CYA25 and CYA37 were floccose with a yellowish green colour. The surface of the lower colonies was yellow on MEA [Fig. 1(A2)] and CYA25 [Fig. 1(C1)], while it was cream-coloured on CYA37 [Fig. 1(C2)].

The microscopic characteristics of the three isolates were also similar. The isolates produced conidiophore with a rough-walled stipe, biseriate, and radiate conidial head with a globose vesicle [Fig. 2(A1)], with diameters ranging from 32.0 μm to 37.0 μm. The conidia were globose to subglobose, with diameters ranging from 4.4 μm to 6.0 μm [Fig. 2(A2)]. Based on the colony and microscopic characteristics, the isolates were morphologically identified as *A. tamarii*, the species description of which was similar to that of *A. tamarii* provided by Klich (2002) and Samson *et al*. (2010).

Molecular identification was performed based on the internal transcribed spacer (ITS) regions, β-tubulin and calmodulin genes. For DNA extraction, mycelia were grown in malt extract broth. Mycelia were harvested after two days of incubation, freeze-dried for 48 h, and subsequently ground to a fine powder in the presence of liquid nitrogen. DNA was extracted using an Invisorb Spin Plant Mini Kit (Stratec Molecular GmbH, Berlin, Germany), following the manufacturer’s instructions. The primers used were based on White *et al*. (1990) for the ITS region, Glass and Donaldson (1995) for β-tubulin, and Hong *et al*. (2005) for calmodulin. Polymerase Chain Reaction (PCR) conditions and cycles were adapted from the methods described by Nur Amalina and Latiffah (2019). A total volume of 25 μL reaction conditions was prepared, containing 4.0 μL of 25 mM MgCl_2_, 0.15 μL of 5U *Taq* polymerase (Promega, Madison, WI, USA), 0.5 μL of 10 mM dNTP mix (Promega), 4.0 μL of 5 mM primers and 0.5 μL of genomic DNA. PCR was performed using a thermocycler (Bio-Rad MyCycler) with initial denaturation at 95°C for 5 min, 30 cycles of denaturation at 95°C for 30 s, annealing at 58°C for ITS region and 56°C for both β-tubulin and calmodulin genes, extension at 72°C for 1 min and final extension at 72°C for 5 min. The products from the PCR were sent to a service provider for DNA sequencing.

The consensus sequences of ITS, β-tubulin, and calmodulin were compared with sequences in GenBank using Basic Local Alignment Search Tool (BLAST) search. Phylogenetic analysis was then performed using Molecular Evolution Genetic Analysis (MEGA7) software (Kumar *et al*. 2016), based on the combined sequences of ITS, β-tubulin and calmodulin. A phylogenetic tree was generated using the maximum-likelihood method. Bootstrap analysis was performed with 1,000 replicates to determine the support for each clade. Three *A. tamarii* reference strains (CBS 133097, CBS 167.63 and CBS 104.13) were included in the analysis. Several *Aspergillus* species section *Flavi* were also included in the phylogenetic analysis, namely *A. pseudotamarii* (CBS 766.97), *A. nomius* (NRRL 13137), *A. oryzae* (CBS 817.72), *A. flavus* (CBS 501.65), *A austwickii* (CBS 135406), *A. aflatoxiformans* (CBS 121.62) and *A. parasiticus* (CBS 100926). *Aspergillus fumigatus* served as the outgroup (CBS 511.64). The species chosen was based on a list of *Aspergillus* species section *Flavi* by Frisvad *et al*. (2019).

Based on BLAST search of ITS, β-tubulin and calmodulin sequences, the isolates showed 99%–100% similarity with the ITS, β-tubulin and calmodulin sequences of *A. tamarii*. The sequences were deposited in GenBank with the following accession numbers: ITS (MF359724, MF359731, MF359732), β-tubulin (MF359728, MF359729, MF359730), and calmodulin (MF359725, MF359726, MF359727). Phylogenetic analysis showed that the three isolates (BPMF17, MF9 and MF24) recovered from banana rot were grouped with the three *A. tamarii* references strains (CBS 133097, CBS 167.63 and CBS 104.13) (Fig. 3). The other *Aspergillus* species section *Flavi* were grouped in separate clades. Thus, all the isolates isolated from banana rot were identified as *A*. *tamarii* which is in agreement with BLAST search results.

The pathogenicity test was conducted using the mycelial plug with wounded and unwounded treatments on *Mas* cultivar. The mycelial plug was taken from the edge of an actively growing mycelium from a 4-day-old culture using a cork borer (0.5 cm). The mycelial plug was placed onto the surface of wounded and unwounded healthy banana fruits. Control banana was inoculated with PDA plug.

For the wounded treatment, the surfaces of banana fruits were cut to approximately 0.5 cm in diameter using a sterile scalpel. The mycelial plugs (0.5 cm) were inoculated onto healthy fruit cut surfaces. The inoculated fruits were incubated at room temperature for 7–10 days and the development of fruit rot was observed daily. Three replicates were prepared for each treatment on the same banana fruit and the pathogenicity test was repeated three times.

The development of fruit rot was determined according to the severity scale based on the area of rotting tissues and severity of the rotting symptom (Table 1) modified from Chavan and Tawade (2012). Symptoms on inoculated banana fruits appeared after 7 days of inoculation, and were similar on all fruits. White mycelia grew from the inoculated area, and the lesions expanded. For isolate BPMF17, the rot symptoms were highly severe (scale 3) following the wounded treatments (Fig. 4). On the unwounded treatments, symptoms were not observed on the inoculated fruits. Rot symptoms were observed on both wounded and unwounded fruits inoculated with isolates MF9 (Fig. 4) and MF24. The symptoms observed were also highly severe (scale 3).

Reisolation of the fungus was performed to fulfill Koch’s postulates. Reisolation of the fungal isolates from the rot lesion showed the presence of the same fungal isolates inoculated on the banana fruits. Morphological identification showed that the re-isolated fungal isolates had the same characteristics as the original isolates isolated from the rot lesion of infected banana.

*Aspergillus tamarii* is a type of spoilage fungi that commonly contaminates different types of nuts, and is occasionally isolated from other food products, including wheat, coffee, spices, dried meat, and fish products (Pitt & Hocking 2009). Thus, as a spoilage fungus, it is likely that *A. tamarii* has the ability to cause rot in banana fruits after harvest. Moreover, *A. tamarii* has been reported as a postharvest pathogen of guava (Valentino *et al*. 2015), and has also been isolated from diseased bananas (Talha Azeem *et al*. 2016), however pathogenicity tests using this fungus have not been conducted. In addition to *A. tamarii*, two species of *Aspergillus*, *A. niger* and *A. flavus* have been reported to be associated with banana fruit rot (Odebode & Janusi 1996). According to Supriya *et al*. (2009), the occurrence of several species of *Aspergillus* in rot lesion including *A. flavus*, *A. fumigatus*, *A. niger* and *A*. *terreus* accelerate banana fruit rot.

The results of the present study indicated that *A. tamarii* was the causal pathogen of banana rot, with more severe infection on wounded fruits. In general, wounds on the surface of fruit crops and other crops are caused by abrasion or cuts during harvesting and handling operations, damage during storage, and poor hygiene during transportation and marketing (Coates & Johnson 1997). Therefore, wounded banana fruits are more susceptible to *A. tamarii* infection. The wound and presence of postharvest pathogens as well as suitable environmental factors may provide conducive conditions for pathogen infection and disease development. On unwounded fruits, the infection was not as severe as that in wounded fruits. These results suggest that *A. tamarii* may produce extracellular enzymes such as xylanases (Dhulappa & Lingappa 2013) to facilitate its penetration into the fruit tissues.

As wounded banana fruits are more susceptible to postharvest pathogens including *A. tamarii*, prevention of wound or injury during harvest and postharvest handling is essential. Wounds are caused by bruises and abrasions during harvesting, insect feeding in the field as well as by chilling and heat injuries during storage. To minimise wounds on banana fruits, several precautions can be taken such as careful harvesting and handling of the produce, controlling pest population in the field, proper packaging and stored at appropriate temperature (Coates & Johnson 1997).

To the best of our knowledge, this is the first report of *A. tamarii* as a causal pathogen of banana fruit rot. Banana fruit rot caused by *A. tamarii* may not solely cause serious damage, compared to the crown rot and anthracnose pathogens of banana. Crown rot and anthracnose are major postharvest disease of fruit crops including banana. Nevertheless, the combined effect of the fruit rot pathogens has the potential to severely infect bananas, which affects their quality and marketability.

**Figure 1:**
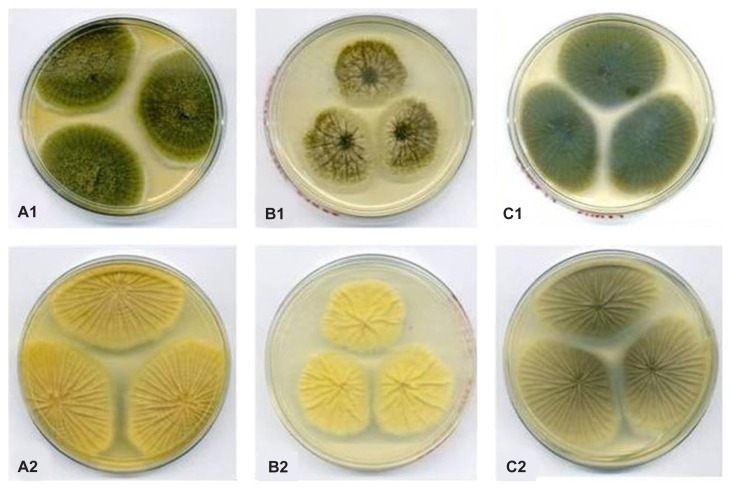
Colony appearance of *A. tamarii* (BPMF17). (A1–A2): Upper and lower colony surfaces on MEA; (B1–B2): Upper and lower colony surfaces on CYA25; and (C1–C2): Upper and lower colony surfaces on CYA37.

**Figure 2:**
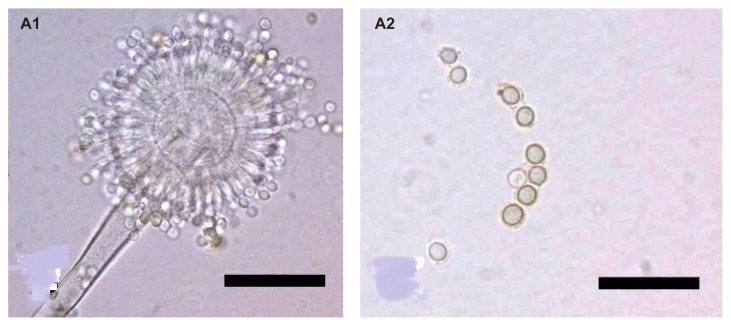
Microscopic observation of conidiophores and conidia of *A. tamarii* (BPMF17). (A1): Globose vesicle, biseriate and radiate conidial head (100×); (A2): Globose to subglobose conidia (100×).

**Figure 3:**
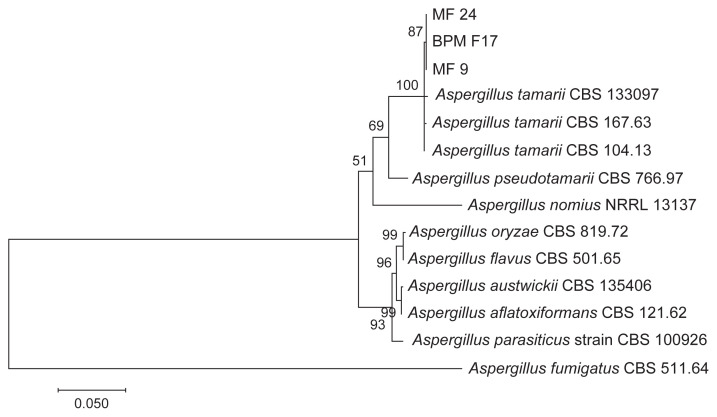
Maximum likelihood tree inferred from combined ITS, β-tubulin and calmodulin sequences of *A. tamarii* from banana rot and *Aspergillus* species section *Flavi*.

**Figure 4:**
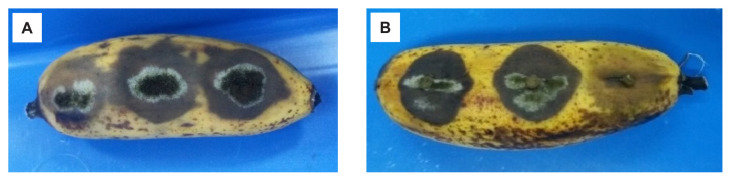
Pathogenicity test of isolate MF9 using mycelial plug on (A) wounded; and (B) unwounded banana showing severe infection.

**Table 1:** Disease severity scale used for disease assessment of banana fruit rot.

Disease scale	Rotting symptom appearance on fruit surface (cm)	Severity
0	0 or ≤ 0.9	No symptom / symptom not obvious
1	−1.9	Slightly severe
2	−2.9	Moderately severe
3	−3.9	Highly severe
